# The human retroviral-like aspartic protease 1 (ASPRV1): From *in vitro* studies to clinical correlations

**DOI:** 10.1016/j.jbc.2024.107634

**Published:** 2024-08-02

**Authors:** János András Mótyán, József Tőzsér

**Affiliations:** Department of Biochemistry and Molecular Biology, Faculty of Medicine, University of Debrecen, Debrecen, Hungary

**Keywords:** ASPRV1, SASPase, aspartic protease, protease, proteolysis, viral protein, skin

## Abstract

The human retroviral-like aspartic protease 1 (ASPRV1) is a retroviral-like protein that was first identified in the skin due to its expression in the *stratum granulosum* layer of the epidermis. Accordingly, it is also referred to as skin-specific aspartic protease. Similar to the retroviral polyproteins, the full-length ASPRV1 also undergoes self-proteolysis, the processing of the precursor is necessary for the autoactivation of the protease domain. ASPRV1’s functions are well-established at the level of the skin: it is part of the epidermal proteolytic network and has a significant contribution to skin moisturization *via* the limited proteolysis of filaggrin; it is only natural protein substrate identified so far. Filaggrin and ASPRV1 are also specific for mammalians, these proteins provide unique features for the skins of these species, and the importance of filaggrin processing in hydration is proved by the fact that some ASPRV1 mutations are associated with skin diseases such as *ichthyosis*. ASPRV1 was also found to be expressed in macrophage-like neutrophil cells, indicating that its functions are not limited to the skin. In addition, differential expression of ASPRV1 was detected in many diseases, with yet unknown significance. The currently known enzymatic characteristics—that had been revealed mainly by *in vitro* studies—and correlations with pathogenic phenotypes imply potentially important functions in multiple cell types, which makes the protein a promising target of functional studies. In this review we describe the currently available knowledge and future perspective in regard to ASPRV1.

The retroviral-like aspartic protease 1 (ASPRV1) is a mammalian protein that had first been identified as a protease (PR) of the human epidermis (EC: 3.4.23.-) being specifically expressed in the granular layer of the skin. Consequently, it was originally named as skin-specific aspartic protease (SASPase) (1, 2), but it is also referred to as 12-O-tetradecanoylphorbol-13-acetate-inducible aspartic proteinase-like protein (TAPS). Both the ASPRV1 and SASPase names are approved and used widely in the scientific literature. *ASPRV1* is one of the human genes that have been domesticated from retrotransposons during vertebrate evolution. The protein expressed from this gene was defined as a retrovirus Gag-like protein as—besides its enzymatic domain—it contains a domain that is homologous to that of the Gag structural domain of the canonical retroviral polyprotein (3, 4, 5). The evolutionary origin was demonstrated by the homology between its Gag-like and protease domains and those of the retrovirus/retrotransposon proteins, as it is described later.

The expression of human ASPRV1 protein was described in the *stratum granulosum* layer of the healthy epidermis by detecting the highest mRNA level in skin and significantly lower levels in the brain (1). Accordingly, data available in the Genotype-Tissue Expression (GTEx) Portal (gtexportal.org) and Human Protein Atlas (proteinatlas.org) (6) databases also show high level of expression in the skin. ASPRV1 was first characterized at protein level in 2005 by Bernard *et al.* at the laboratories of L’Oreal and Galderma (1). It was found to be localized in the nucleus and cytoplasm of epidermal granular layer cells (1, 7). Later, Whittaker Hawkins *et al.* analyzed samples from patients both with and without multiple sclerosis (MS) and found that the mRNA of human ASPRV1 is highly abundant in blood neutrophils in the steady state, but not in other cell types such as B cells, monocytes, and unfractionated mononuclear cell. In addition, the mRNA level was higher in lesions of postmortem brain samples of patients with severe MS as compared to the samples obtained from normal white matters or from lesions of mild and moderate MS (8). Database information (Human Protein Atlas) also imply that the expression of ASPRV1 is not limited to the skin and is high in kidney cells and placenta. Other retroviral-like proteins are also exhibit high placental expression; such as paternally expressed gene 10 (PEG10) protein (9, 10).

## Protein name

The human ASPRV1 was first identified as a skin-specific enzyme in 2005, thus, it has primarily been named as skin-specific aspartic protease (SASPase) (1). Later, the ASPRV1 name was also introduced (11), both SASPase and ASPRV1 names are commonly used in the literate. As it is discussed below, there are three main forms of ASPRV1 having different molecular weights of 37, 28, and 14 kDa, which are still distinguished from each other by using SASPase-based names (SASP37, SASP28, and SASP14).

In order to avoid inconsistency and better reflect the common name of the gene, we recommend the unification of the nomenclature and the use of ASPRV1 rather than SASPase. The ASPRV1 name reflects the origin, and does not limit the tissue specificity to the skin. In accordance with this, we recommend the use of the ASPRV1-based nomenclature to distinguish the three protein forms and introduce here the ASPRV1-37, ASPRV1-28, and ASPRV1-14 names to replace the respective SASP37, SASP28, and SASP14 ones.

## Synthesis, domain organization, sequence, and structure of ASPRV1

**Figure 1:**
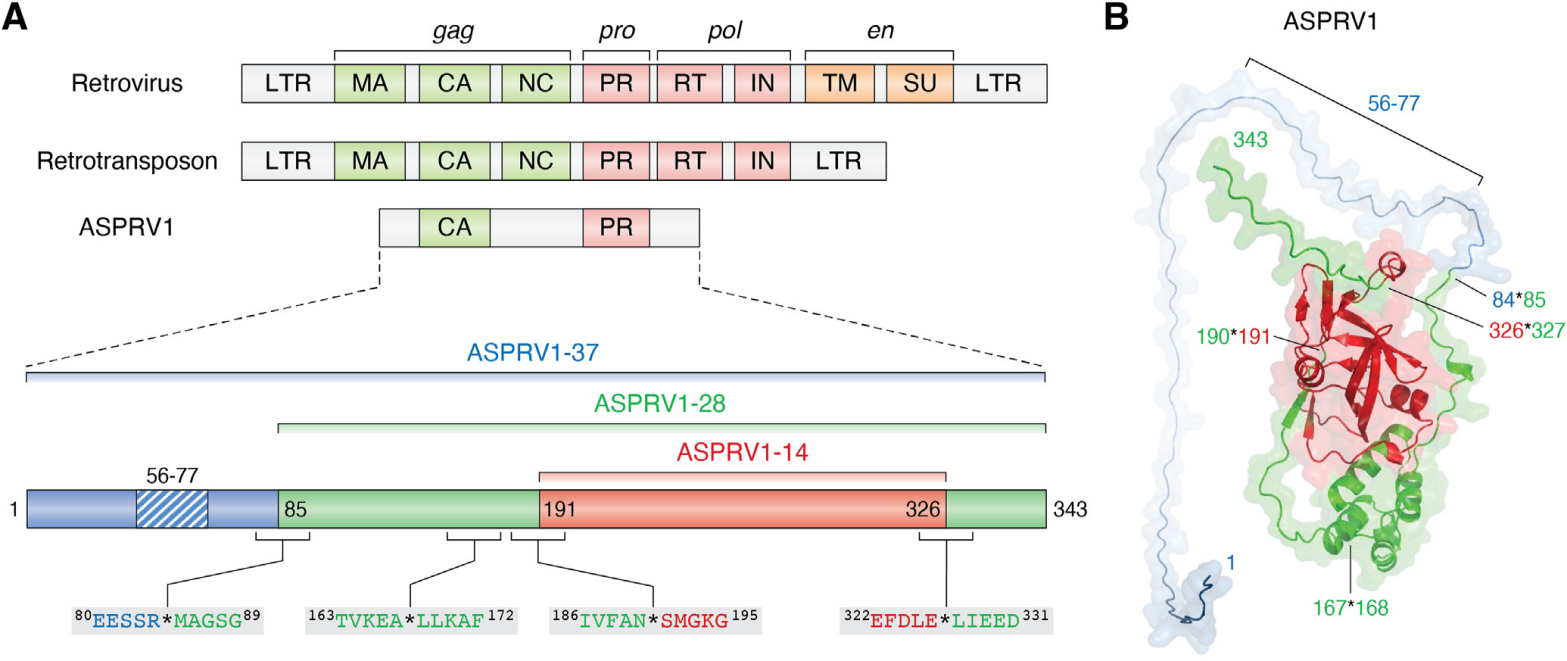
**Domain organization of human ASPRV1.***A*, organization of retroviral and retrotransposon genomes and domain organization of ASPRV1 protein. Matrix: MA, capsid: CA, nucleocapsid: NC, protease: PR, integrase: IN, reverse transcriptase: RT, transmembrane: TM, surface: SU, long terminal repeat: LTR. The schematic representation of ASPRV1’s domain organization is also shown. The hydrophobic region close to the N terminus is *striped*, the sequences of the autoproteolytic cleavage sites are also indicated. *B*, the structure of the full-length ASPRV1 protein is represented based on a model structure (AlphaFold: AF-Q53RT3-F1) by using the color code of domains in figure part *A*. The hydrophobic region and the self-processing sites are shown by *arrows*. ASPRV1, retroviral-like aspartic protease 1; LTR, long terminal repeat.

The canonical genomes of retroviruses, like myeloblastosis-associated virus (MAV, also referred to as AMV) and HIV-1 consist of four main genes: the *gag*, *pro*, *pol*, and *env* (Fig. 1). The *gag* gene encodes the structural Gag proteins (matrix, capsid, and nucleocapsid), the *pro* gene for the protease domain, the *pol* gene codes for the enzymatic domains (reverse transcriptase and integrase), while the surface envelope proteins are translated from the *env* gene. These genes code for the structural proteins and replication enzymes, the functional domains are synthesized as a part of long Gag and Gag-Pro-Pol polyproteins. The *ASPRV1* gene exhibits characteristic differences, compared to the genomes of retroviruses and retrotransposons; it is significantly shorter due to the lack of most protein-coding regions and long terminal repeats (3, 4, 12, 13) (Fig. 1*A*). The ASPRV1 *gene* codes for a protein that contains two globular proteins that are homologs of the retroviral capsid and protease, whose characteristics are described in details below.

The protease domain of the retroviral polyprotein is encoded by the *pro* gene which can be either in or out of frame with the *gag* and *pol* genes. In case of the MAV, the *pro* is in frame with *gag* and *pol*; thus, the protease and the structural proteins (matrix, capsid, and nucleocapsid) are also translated as part of the Gag-Pro polyprotein. In other cases, these genes are not in the same ORF, and there is a termination codon at the 3′ end of *gag*. In order to translate the *pro* and *pol* genes, the termination codon can be bypassed by using two different mechanisms. The first (exemplified *e.g.* by moloney murine leukemia virus) is the so-called readthrough-suppression (or stop codon-suppression), in which the termination codon of *gag* is misread as a sense codon, resulting in the continuation of the translation from *pro-pol* reading frame. The second mechanism is the ribosomal frameshifting, which is utilized by most retroviruses, including HIV-1. In this mechanism, there is a movement of the ribosome toward 5′ direction during translation (−1 frameshift), thus, the translation is not terminated at the C-terminus of the Gag, rather, the frameshifting enables the continuation of the translation from the reading frame of *pro-pol*, *i.e.* the synthesis of the Gag-Pro-Pol polyprotein containing the viral PR (14, 15).

The translation mechanisms are considered to regulate the protease activity. The viruses using stop codon-suppression or ribosomal frameshifting produce the PR in remarkably lower amount than the Gag. In the case of HIV-1, the efficiency of the ribosomal frameshifting is very low (5–10%), thus, the Gag proteins are produced in much higher amount than the PR (16). In contrast to this, the viruses in which the *pro* gene is in frame with gag (*e.g.* MAV); the Gag and PR therefore are synthesized in equivalent amount.

It has been revealed that the −1 ribosomal frameshifting is not unique to retroviruses, some full-length Gag-like proteins are also translated by using this mechanism, such as PEG10 and the retrotransposon Gag-like protein 3 (RTL3) (9, 17). Interestingly, the efficiency of PEG10’s frameshifting was found to be unusually high (∼60%) (17), indicating that the ratio of the PR (translated from the second ORF) and the structural domains (translated from the first ORF and corresponding to the retroviral Gag) is higher compared to HIV-1. Unlike most retroviruses (including HIV-1), a −1 ribosomal frameshift mechanism is not utilized for the synthesis of the full-length ASPRV1, its Gag-like and PR domains are synthesized in equimolar amount. ASPRV1 belongs to that group of retroviral-like proteins which are synthesized without using −1 ribosomal frameshifting, that is used by only few domesticated proteins (PEG10 and RTL3) (3, 4, 5, 13).

Human ASPRV1 consists of 343 amino acid residues. The full-length precursor protein (1–343) has a 37 kDa molecular weight, it is referred to as ASPRV1-37 (based on the SASPase-based nomenclature it is SASP37). The shorter forms have approximately 28 and 14 kDa molecular weight, therefore, they are referred to as ASPRV1-28 and ASPRV1-14, respectively (the respective SASPase name are SASP28 and SASP14) (1). A Gag-like domain (100–172) being a homolog of the retroviral/retrotransposon capsid (CA) protein is localized in the central part of ASPRV1-37, it is present in ASPRV1-28, as well, but absent from the shortest ASPRV1-14 form. The C-terminal region of ASPRV1 contains its protease domain that is homologous to retroviral proteases. This enzymatic domain is present in each form of ASPRV1, and it is responsible for the *cis*- and *trans*-activity, as well (Fig. 1). The ASPRV1-37 precursor undergoes self-proteolysis, the autoproteolytic cleavage between 84th and 85th residues release the ∼28 kDa proform (ASPRV1-28). This proform is further processed; it is cleaved at the N and C termini of the protease domain. The limited proteolysis at 190∗191 and 326∗327 cleavage sites release the aspartic protease domain that has ∼14 kDa molecular weight (ASPRV1-14) (Fig. 1, *A* and *B*).

The structure of the full-length ASPRV1, as well as that of its individual domains have not yet been determined experimentally, therefore, the structural characteristics were only investigated by using model structures. Homology modeling was used by some studies to estimate the tertiary or quaternary structure of the protease domain only (1, 7, 18). A model structure of this domain is available in the SWISS-MODEL Repository (19) as well as in AlphaFold Protein Structure Database (20). The structural features of the Gag-like domain has not been investigated by any study, to our best knowledge, the only structural coordinate that contains the Gag-like domain was prepared by AlphaFold.

The N-terminal region of ASPRV1 contains a short hydrophobic region (F56-E77) which is absent from ASPRV1-28 and −14 protein forms, because it is released from the precursor during the autoproteolysis. The vast majority of the publications refer this region to be putatively transmembrane, but database information (*e.g.* Human Protein Atlas) and literature data (1, 7) also imply that ASPRV1 protein has mainly cytoplasmic and occasionally nuclear expression. To our best knowledge, there is no experimental evidence (*e.g.* by immunostaining) regarding the localization of ASPRV1 on the cell surface, and association with intracellular membranes was not reported so far. This is the reason why the short region close to the N terminus of ASPRV1 is still referred to only as “putatively transmembrane” in the literature (1, 2, 7, 8, 21). The DeepTMHMM 1.0 algorithm was developed for the prediction of transmembrane helices in proteins (https://services.healthtech.dtu.dk/services/DeepTMHMM-1.0) (22). We have analyzed the sequence of ASPRV1 (UniProtKB: Q53RT3) by this online tool, which implied relatively higher hydrophobicity for the F56-E77 region, but it was not predicted to be transmembrane. Analysis of the sequence by ExPASy ProtScale online tool (23) also revealed that this region is more hydrophobic than others of the protein. We describe thus this region as hydrophobic rather than transmembrane, although, it might be responsible for the association of ASPRV1 to membranes or mediating protein-protein interactions. It is important to note that the biological functions of ASPRV1 are likely to be not limited to the intracellular space; it is believed to contribute to the proteolytic processing of (as yet unknown) extracellular substrates (8). A recent analysis identified ASPRV1 in nasal epithelial lining fluid as an extracellular protease (24), but its function in the airway remain to be determined by future studies.

### Gag-like domain of ASPRV1

**Figure 2:**
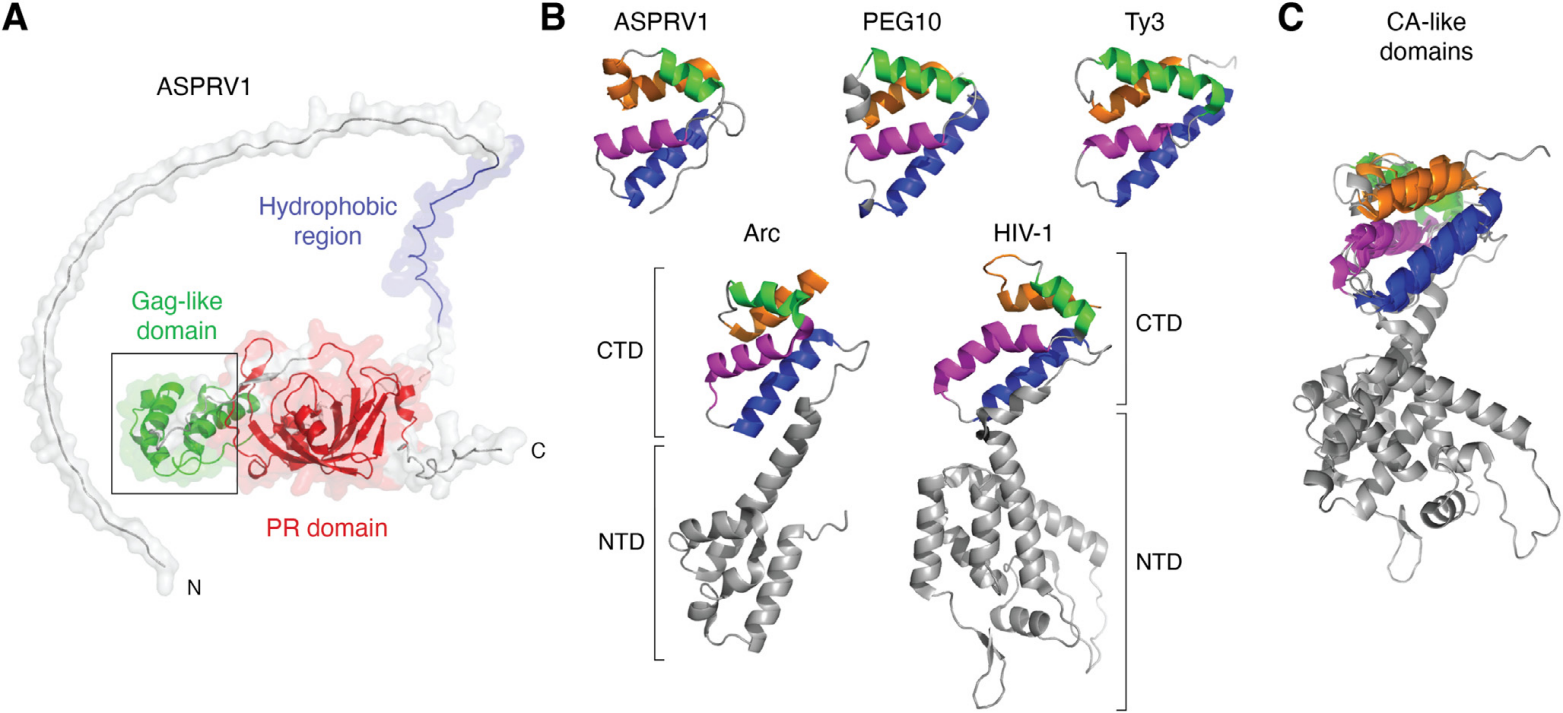
**Comparison of capsid and capsid-like domains.***A*, the structure of the full-length ASPRV1 is shown based on a model structure (AlphaFold: AF-Q53RT3-F1) that was downloaded from AlphaFold Protein Structure Database. The hydrophobic region has *blue*, the PR domain *red* while the Gag-like domain *green* color, corresponding to domain coloring used in Figure 1. *B*, the CA-like domains of human PEG10 (AlphaFold: AF-Q86TG7-F1) and human ASPRV1 (100–172; AlphaFold: AF-Q53RT3-F1) are shown based on a model structure, while the structures of human Arc (212–356) (PDBID: 7R23) (deposited by Markusson S. and Kursula P.) and Ty3 retrotransposon (PDBID: 6R22) (131) are shown based on their crystal structures. For comparison, a retroviral CA domain is also shown, the figure represents the HIV-1 CA based on a crystal structure (PDBID: 3NTE) (132). The corresponding α-helices have the same color (*blue*, *purple*, *green*, and *orange*). The N- and C-terminal domains are labeled as NTD and CTD, respectively. *C*, superposition of the CA-like domains of Ty3 retrotransposon, human ASPRV1, Arc and PEG10 proteins as well as the HIV-1 CA. ASPRV1, retroviral-like aspartic protease 1; CTD, C-terminal subdomain; NTD, N-terminal subdomain; PEG10, paternally expressed gene 10; PR, protease.

Multiple studies have already been conducted on domesticated retroelement-derived genes aiming to compare the structural arrangements of the proteins synthesized from these genes. Interestingly, the findings of these studies are controversial. Some of the studies imply that ASPRV1 lacks a CA-like domain (13, 25), while others revealed the existence of this domain (3, 4). A recent review also classifies ASPRV1 into the group of CA-like domain-containing proteins (5). In agreement with this, the structural comparison we represent in Figure 2 also confirms the presence of the C-terminal subdomain (CTD) of the CA-like domain in ASPRV1, nevertheless, the N--terminal subdomain is indeed missing.

Information about the gag-like domain of ASPRV1 is limited. It shows high structural similarity to the CA domain of Ty3 retrotransposon and the CA-like domains of Gag-like homolog proteins such as human activity-regulated cytoskeleton-associated protein (Arc) as well as PEG10 and PEG11 proteins (3, 4). The CA-like domain of ASPRV1 is short and highly similar to the CTD of human Arc and PEG10 proteins’ CA-like domain, and to the CTD of HIV-1 CA protein (Fig. 2). A number of capsid-forming Gag-homologs is known to have the ability for assembly into a capsid-like oligomer, such as PEG10, Arc, the retrotransposon-like protein 1 (RTL1, also referred to as PEG11), modulator of apoptosis 1, or paraneoplastic antigen Ma proteins, while mouse ASPRV1 was found to lack the ability to generate extracellular virus-like particles (4). The high similarity of human and mouse orthologues implies that the human protein is also unable to self-assemble. In addition, ASPRV1 lacks any nucleic acid-binding motifs, therefore, it cannot specifically bind mRNAs, unlike PEG10 (4).

Filaggrin 2 (FLG2) protein was found to interact with ASPRV1 *via* their structurally similar domains that has been referred to as single interacting domain (SID) in the case of both proteins (21). The so-called SID domain of ASPRV1 (97–169) is located prior to the protease domain in between the two main autoproteolytic sites (84∗85 and 190∗191), preceding the PR domain (Fig. 1), thus, it corresponds to the CTD of the CA-like domain (100–172). Although, ASPRV1—as a Gag-like protein—is defective for the formation of virus-like capsids, its CA-like domain plays a crucial role in the regulation of protein function *via* mediating protein-protein interactions, as discussed below in the article. The SID domain is present only in the ASPRV1-37 and ASPRV1-28 forms, thus, it can contribute to the regulation of precursor forms’ processing and the formation of mature ASPRV1-14.

### Proteolytic enzymes and protease domain of ASPRV1

ASPRV1 contains an enzymatic domain in its C terminus. The shortest enzyme form (ASPRV1-14) consists only of this catalytically active aspartic protease domain. Accordingly, ASPRV1 belongs to the human degradome which includes the complete set of proteases present in the human body. The proteolytic enzymes are classified based on their active site residues and catalytic mechanism. The groups of the serine, cysteine, and metalloproteases are the largest, followed by the aspartic and threonine protease groups. Information on the proteolytic enzymesis available in a protease-specific knowledgebase; in the MEROPS database (26).

ASPRV1 is a member of the aspartic PR family (EC 3.4.23). All of these enzymes are endopeptidases, *i.e.* cleave peptide bonds within their (poly)peptide substrates. The hydrolysis of the peptide bonds is catalyzed by two aspartate residues, a catalytic water molecule that acts as a nucleophile is also involved in the catalysis. Based on the Mammalian Degradome Database (http://degradome.uniovi.es.), the number of human aspartic proteases is 21 (27, 28).

The cellular aspartic PRs of eukaryotes are classified into two families, the pepsin-like family (A1) and the so-called retropepsin family (A2) (29). The most characteristic members of the pepsin-like family are cellular enzymes such as pepsin, renin, chymosin, as well as cathepsin D and E. The retropepsin family includes PRs of retroviruses and retrotransposons, such as that of the HIV-1 and the Ty1 retrotransposon of budding yeast *Saccharmoyces cerevisiae*, respectively. In addition, retroviral-like PRs of vertebrates also belong to this enzyme family, such as DNA damage inducible 1 homolog 1 and 2 proteins (Ddi1 and Ddi2), PEG10, PEG11, as well as ASPRV1 (30).

The pepsin-like PRs are monomeric and the catalytically active enzyme is bilobal. In contrast to this, the retropepsins are homodimers in their catalytically active forms. Both pepsin-like enzymes and retropepsins exhibit high structural similarity, the monomers of retropepsins resemble the N- and C-terminal lobes of the pepsin-like enzymes (31). The aspartates of the catalytic dyad are provided by the N- and C-terminal lobes of the single-chain pepsin-like enzymes, while each monomer contains only one of the catalytic residue; therefore, homodimerization is a prerequisite for catalysis. The intramolecular two-fold symmetry is a consequence of the evolution of the bilobal proteases by gene duplication, fusion, and divergence through mutation events. The evolution of PRs by gene duplication is not restricted to the aspartic PRs, similar evolution mechanism was suggested *e.g.* for the serine PR chymotrypsin, as well (32). A common feature of the pepsin-like PRs and the retropepsins is that they can be inhibited by the hexapeptide pepstain A. Pepstain A is considered as a general inhibitor of aspartic proteases, although interestingly it was unable to inhibit some of the retroviral-like aspartic PRs such as Ty1 (33) and PEG10 (34)), and it is not a potent inhibitor of ASPRV1 (1, 18).

**Figure 3:**
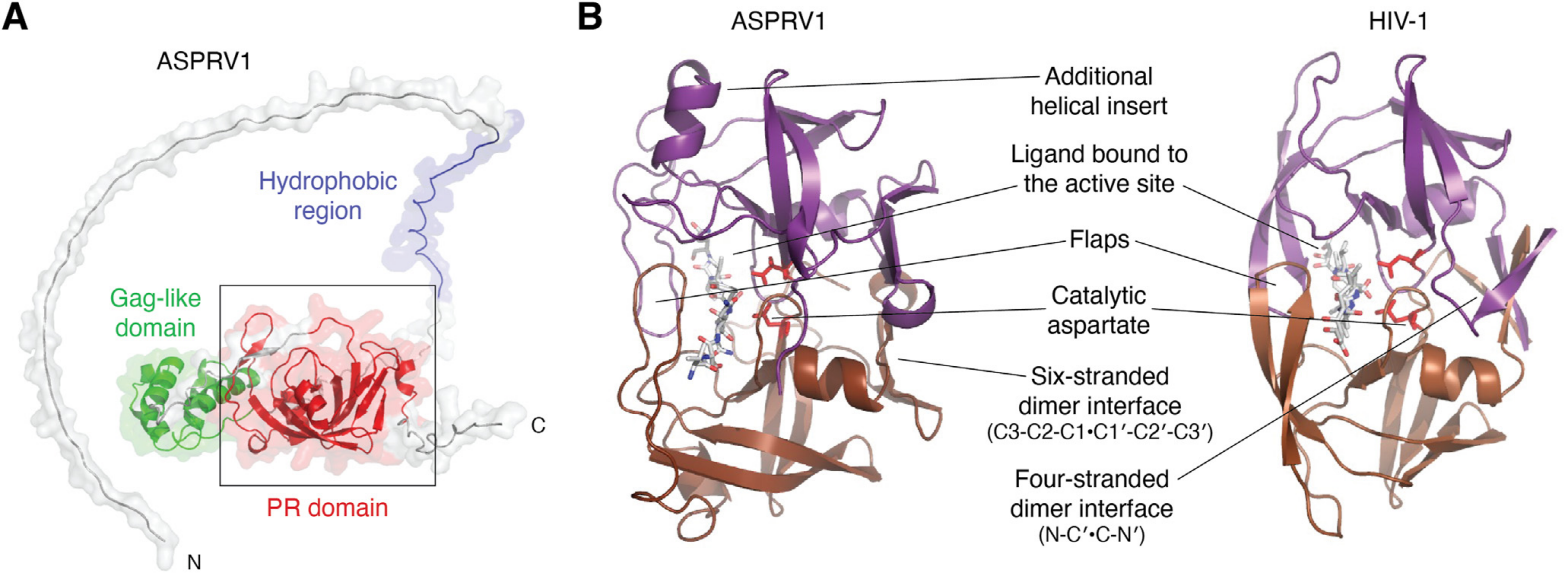
**Structure of ASPRV1-14 and HIV-1 PR.***A*, the structure of the full-length ASPRV1 is represented based on Figure 2. *B*, structures of ASPRV1-14 and HIV-1 PR are compared. The quaternary structure of the ASPRV1’s protease domain (ASPRV1-14) complexed with an oligopeptide substrate (VSQNY∗PIVQ) is represented based on a homology model (18). HIV-1 PR is shown based on a crystal structure of the enzyme complexed with acetyl-pepstatin inhibitor (PDBID: 5HVP) (133). The most important regions and sites are labeled, the flaps have closed conformation in both cases. ASPRV1, retroviral-like aspartic protease 1; PR, protease.

The protease domain of ASPRV1 shares high structural similarity to HIV-1 protease (Fig. 3). In contrast to some cellular homolog retroviral-like PRs, such as the Ddi1 and Ddi2 (35, 36, 37), ASPRV1 contains no ubiquitin-like or helical domain of Ddi1 domains. Similar to retroviral PRs, the catalytically active enzyme is formed by two identical subunits (Fig. 3). The homodimer is stabilized mainly by the “fireman’s grip” interactions formed between the Ser residues of its D-S-G-A active site motif (38) and by the dimerization interface consisting of six nonalternating β-strands (Fig. 3). The replacement of the catalytic aspartate by alanine was found to inactivate aspartic proteases such as HIV-1 PR (38). Bernard *et al.* introduced mutations to the D-S-G-A active site motif and found that both D212A and D212E mutant ASPRV1 lost its ability for self-proteolysis (1), and the D212N mutation also abolished the proteolytic activity (39). In agreement with this, studies on the mouse homolog protein also revealed that mutation of the catalytic aspartate (D212A) inactivates the protease (2). These findings proved that ASPRV1 belongs to the aspartic protease family and that the aspartate residue in its active site motif is essential for the catalytic activity, similar to the retroviral homodimeric aspartic proteases. To our knowledge, other ASPRV1 mutant proteins that represent nonnatural sequence variants have not yet been studied.

To date, the structural characteristics of ASPRV1 have only been explored at the level of its protease domain. Each study used homology modeling for structure building (1, 7, 18). Despite the relatively low sequence identity, the structures of retroviral and retroviral-like PRs are highly similar (40); thus, the protease domain of ASPRV1 was predicted to share its overall fold with the retroviral and retroviral-like PRs. Of the retroviral PRs, the xenotropic murine leukemia virus-related virus (XMRV) PR (41) exhibits the highest sequence identity with the protease of ASPRV1. Of the cellular retroviral-like enzymes, the ASPRV1 PR is highly similar to the human Ddi1 (2I1A.pdb) (42), Ddi2 (4RGH.pdb) (35) and PEG10 (34) PRs.

The dimer interface of the homodimeric ASPRV1-14 is exclusively formed by C-terminal β-strands of the monomers, the β-sheet at the dimer interface was predicted to be six-stranded and contains no alternating β-strands (18). This interface organization closely resembles that of the retroviral XMRV PR (41, 43), the Ty1 retrotransposon PR of *S. cerevisiae* (33), and human retroviral-like PRs, such as Ddi1 (42), Ddi2 (35), and PEG10 (34). A dimer interface that is formed by 3-3 C-terminal β-strands of both monomers was proposed to be a common feature of retroviral-like PRs (40).

The currently available structural models (1, 18) imply that the flap conformations of ASPRV1 are similar to those of the retroviral PRs and cover the active site (Fig. 3). In contrast to this, Ddi1 and Ddi2 retroviral-like PRs were found to have structurally different flaps, which do not cover the active site, and exhibit a conformation that is unusual for retroviral PRs (35). It is known that the flaps of HIV-1 PR are flexible and can exhibit opened and closed conformations. The opened conformation enables the binding of the ligand to the active site (and the release of the cleavage products, as well), while in the closed conformation, the flaps wrap around the ligand (either substrate or inhibitor) (44). The flaps are represented in their closed conformation in Figure 3. The unique flap conformations of the Ddi1 and Ddi2 PRs imply that the retroviral-like proteases—including ASPRV1—might have unique flap conformations, thus, the structural requirements of substrate recognition might be slightly different from those of the retroviral PRs. In addition, interactions between the flaps contribute to the stabilization of the homodimeric HIV-1 PR (40), but the corresponding intermonomeric interactions are missing from the Ddi1 and Ddi2 PRs where the active site is not covered by the flaps. The reliability of homology models hinges on the currently available template structures. Future experimental studies may elucidate whether the flap conformations of ASPRV1 PR are more akin to retroviral-like PRs.

The structures of the Gag-like domains have been resolved by X-ray crystallography solely for Arc (45), PEG10, and modulator of apoptosis 1 proteins (46); the experimental determination of the structure of ASPRV1’s CA-like domain remains to be conducted.

## Enzymatic and functional characteristics of ASPRV1 protease

Expression of the full-length precursor (*i.e.* ASPRV1-37) form in *Escherichia coli* cells was found to be insufficient due to the presence of the hydrophobic region close to its N terminus (1); therefore, the enzymatic characteristics have only been studied *in vitro* at the level of the WT and mutant shorter protein forms (ASPRV1-28 and ASPRV1-14) (1, 7, 18, 21, 47). Enzymatic studies were performed for mouse SASP32 and SASP15, as well (2), but most data are available for the human protein. The suggested nomenclature can be applied to the mouse protein, designating the full-length proteins (SASP32) as ASPRV-32, and mature protein (SASP15) as ASPRV1-15.

ASPRV1 was found to undergo self-proteolysis (1). The release of ASPRV1- ASPRV1-14 from the ASPRV1-7 and ASPRV1-28 protein forms *via* cleavages at N and C termini of ASPRV1-14 was found to increase the catalytic activity (18). Targeted mutations of the N-terminal cleavage site (A189K/N190I) were found to decrease the efficiency of the self-proteolysis (18). Autoactivation of ASPRV1 *via* limited proteolysis resembles that of the retroviral polyproteins, the molecular mechanism of this self-processing has already been delineated for HIV-1 PR (48, 49, 50). The first cleavage (at the N terminus of the protease domain) during autoactivation is intramolecular, followed by processing at the C terminus *via* intermolecular cleavage. The most recent findings revealed that the sequential order of the cleavages is similar in the case of the severe acute respiratory syndrome coronavirus 2 (SARS-CoV-2) main protease (Mpro) (51). This self-activation mechanism is characteristic for the PRs of RNA viruses (51), but we assume that domesticated eukaryotic PEG10 and ASPRV1 PRs are also activated by N-terminal intramolecular and C-terminal intermolecular cleavages, and this activation mechanism might be potentially a common feature of both viral and cellular PRs that are activated from a multidomain precursor.

The optimal pH for the protease activity of human ASPRV1 was found to be 6.3 in META (18) and 5 in acetate buffer (1), while highest activity for the mouse enzyme was observed at 5.8 in phosphate buffer (2). The slightly acidic pH resembles the environment of the *stratum corneum* that is more acidic, compared to the deeper neutral layers of the skin. ASPRV1-14 (fused with N-terminal GST) showed the highest activity in the presence of 2 M NaCl (52), and the higher ionic strength (0.7 *versus* 0.15 M NaCl) boosted the activity of the mouse ortholog (ASPRV1-15) (2).

The temperature optimum of ASPRV1 has not yet been investigated in details. The protease activity was measured at 37 °C in all of the published studies, and this temperature was found to be sufficient for this cellular enzyme.

It is known that dimerization is a prerequisite for the activity of retroviral aspartic PRs, therefore, the stability of the homodimer can be studied by measuring the activity in the presence of urea. Dimerization of the mouse ortholog ASPRV1-15 was observed while it was purified by gel filtration (2). The human ASPRV1 (GST-fused ASPRV1-14) was found to exhibit relatively lower stability at higher urea concentrations. Determination of the urea concentration that results in 50% loss of enzyme activity (urea dissociation constant) revealed that the ASPRV1 has relatively lower dimer stability, compared to HIV-1 PR (18), and the urea dissociation constant was similar to that of the XMRV (43) and Ty1 retrotransposon PRs (33). The difference can be explained in part by the different dimer interface organizations of these PRs. Homodimeric retroviral PRs with dimer interface consisting of alternating dimer interface exhibit a higher density of intermonomeric contacts, providing higher stability for the dimeric enzymes *in vitro* (40).

## Substrates, cleavage site sequences, and specificity

Interactions between the proteases and their substrates are commonly described by using the nomenclature that was introduced by Schechter and Berger in 1967 (53). According to this nomenclature, the active site of the protease is divided into subsites; each site is responsible for the binding of an amino acid residue of the substrate. Substrate residues harboring the cleaved peptide bond are designated as P1 and P1′ residues, the numbering of the other substrate residues increases toward the N- (P2, P3, P4, and so on) and C-terminal directions (P1′, P2', P3′ and so on). The substrate binding subsites of the active sites are also numbered based on the corresponding residues occupying them (*e.g.* S2, S1, S1’, and S2′). The active sites of retroviral PRs interact mainly with eight substrate residues that are covered by the flaps (P4-P4′) (15, 54, 55). Analysis of HIV-1 and human T-cell leukemia virus type 1 PRs revealed that surface residues of the enzymes also contribute to substrate binding, the binding surface which enables interactions with P12-P5 and P5′-P12′ residues was referred to as substrate-groove (or S-groove) (56). The substrate-groove was studied *in vitro* in case of the Ty1 retrotransposon PR, as well (33), however, the role of protease surface-residues to the substrate recognition has not yet been confirmed for ASPRV1.

Profilaggrin (pro-FLG) is the only known natural substrate of the mature ASPRV1-14, additional natural substrates have not been identified to date. The functional importance of the ASPRV1’s protease activity in the moisturization of the skin is described as follows. ASPRV1 is known to be a member of the epidermal proteolytic network (57) where it contributes to the limited proteolysis of the pro-FLG (47, 58). Pro-FLG is a large molecule (>400 kDa) which consists of repeated FLG units connected by short linkers. It is processed by multiple PRs, one of which is ASPRV1. ASPRV1 cleaves the pro-FLG at the linker sequences between the FLG units. The consensus cleavage site sequence in the human pro-FLG molecule (UniProtKB: P20930) is GSFLY∗QVSTH (Table 1), although, few linker sequences of the pro-FLG molecule contain different residues in some positions (P3-Ser, P2-Ile, and P5-Arg and P4′-Ser). The monomeric FLG units are released from pro-FLG *via* the cleavage by ASPRV1, the monomers undergo further processing (breakdown by other proteases such as cysteine protease calpain 1 and caspase 14) and additional modifications (such as deamination, cyclization, and citrullination). Finally, a mixture of amino acids is produced which helps maintain the hydration of *stratum corneum* and thus designated as “natural moisturizing factor (NMF)” (59, 60, 61, 62).

**Table 1:** Substrates and cleavage site sequences of human ASPRV1

Investigated substrates and cleavage sites	Number	Substrate	Cleavage site sequence	Reference
Natural protein substrate	1	Filaggrin	GSFLY∗QVSTH	(47)
Autoprocessing sites in ASPRV1 protein	2	ASPRV1-28 N terminal	EESSR∗MAGSG	(1)
	3	Alternative cleavage site	TVKEA∗LLKAF	
	4	ASPRV1-14 N terminal	IVFAN∗SMGKG	
	5	ASPRV1-14 C terminal	EFDLE∗LIEED	
Other protein substrates	6	Insulin	SHLVE∗ALYLV	
	7	Insulin	LVEAL∗YLVCG	
	8	Insulin	VEALY∗LVCGE	
	9	Casein	unknown	
Other peptide substrates	10	Synthetic peptide	LFAN∗SMGK	(2)
	11	HIV-1 MA/CA P2-Leu mutant	VSQLY∗PIVQ	(18)
	12	HIV-1 MA/CA P2-Phe mutant	VSQFY∗PIVQ	
	13	HIV-1 MA/CA P2-Val mutant	VSQVY∗PIVQ	
	14	HIV-1 MA/CA P2-Ala mutant	VSQAY∗PIVQ	
	15	Synthetic peptide	QIDRIMEK^#^	(21)
Peptide substrates known to be not processed	16	HIV-1 MA/CA WT	VSQNYPIVQ	(18)
	17	HIV-1 MA/CA P2-Lys mutant	VSQKYPIVQ	
	18	HIV-1 MA/CA P3-Val mutant	VSVNYPIVQ	
	19	HIV-1 MA/CA P3-Asp mutant	VSDNYPIVQ	

The known substrates and cleavage sites are shown in the table, and the cleavage sites that were found to be not processed are also indicated. Asterisk indicates cleavage position within the sequences. MA/CA indicates the matrix/capsid cleavage site of HIV-1 polyprotein. ^#^ Substrate containing unknown cleavage position. Based on the BLAST analysis, the sequence of this peptide is unique for human synaptosomal-associated protein 25 (SNAP25), but it has not been proved experimentally whether this protein is a physiological substrate of ASPRV1.

**Figure 4:**
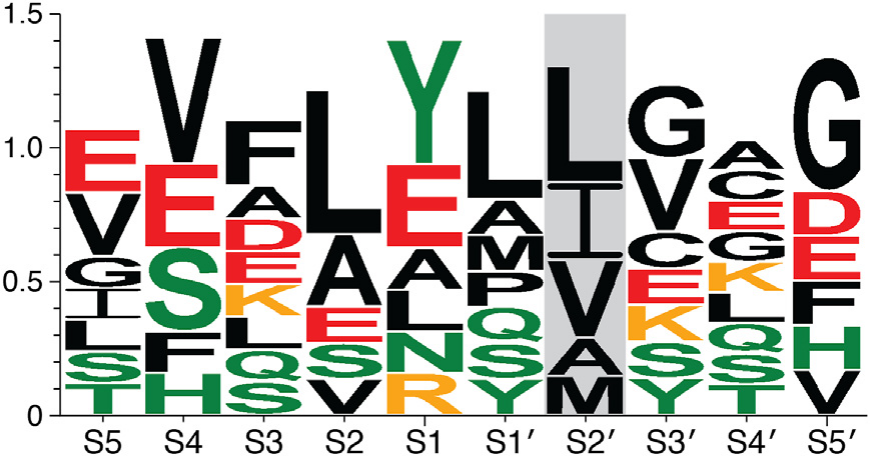
**Sequence logo based on cleavage sites of human ASPRV1.** The figure was prepared based on the cleavage site sequences (sequence 1–8. and 11. in Table 1), by using WebLogo3 (http://weblogo.threeplusone.com) (134). Color code: *red*, negatively charged (D and E); *orange*, positively charged (K and R); *green*, polar (S, T, N, Q, Y, and H); *black*, hydrophobic. Residues binding to S2′ site are framed. ASPRV1, retroviral-like aspartic protease 1.

Most cleavage positions were determined by *in vitro* studies, such as by the primary identification of ASPRV1’s substrates (1) and by the investigation of P2 and P3 amino acid preferences that was performed using a limited set of peptide substrates representing HIV-1 MA/CA cleavage site (18) (Table 1). Based on the currently available cleavage site sequences ASPRV1 exhibits a strong preference for hydrophobic residues in P2 and P2′ positions, and for mainly nonhydrophobic residues in P4-P3 and P3′-P4′ positions (Fig. 4). Similar to HIV-1 PR (15), the polar residues are prevalent at these positions in the known ASPRV1 cleavage sites. Polar resides may also occupy the S2 site (such sequences are the N-terminal and alternative autoproteolytic cleavage sites of ASPRV1-28), but in these cases, the S2′ site binds hydrophobic residues. The S2′ site shows the most rigid specificity, the P2 positions of the known substrates are occupied exclusively by residues containing hydrophobic side-chain (Fig. 4). Various residues can be found in P1 position, including hydrophobic and charged residues, but primarily polar residues bind to the S1 site in the known cleavage sites.

The retroviral protease cleavage sites can be distinguished based on the residues that occupy the S1 and S1′ sites. Type 1 cleavage sites have an aromatic residue and Pro in P1 and P1′ positions, respectively, while type 2 sequences contain hydrophobic residues (excluding proline) at the site of cleavage (15). Neither the autoproteolytic sites of ASPRV1 not its cleavage sites in filaggrin (Table 1, substrate: 1, 2, 4, and 5) represent type 1 or type 2 retroviral protease cleavage sites, as none of these sequences contain proline in P1′ position and the P1 residues are nonhydrophobic. The currently known substrates (Table 1) do not provide sufficient information about the P1′ specificity of ASPRV1. Analysis of additional cleavage site sequences is necessary to reveal sequence context-dependent nature of ASPRV1’s substrate recognition, *i.e.* how interaction of the internal substrate residues determines the cleavage rates. Studies on HIV-1 PR have already shown that the internal residues of the cleavage sites influence the positions of the surrounding side-chains and/or the space being available for their binding. For example, the nature of the P1′ side-chain can affect the enzyme-substrate interactions at S2, S1, and S2’ sites, potentially interfering with efficiency of binding and cleavage (63, 64).

The existence of the alternative cleavage site within ASPRV1-28 (prior to ASPRV1-14 protease domain) was described in the first report on the ASPRV1 (1); however, the functional importance of this cleavage site has not yet been elucidated. Insulin and casein are also widely applied as general substrates of proteolytic enzymes, but it is unlikely that the proteolysis of these molecules by ASPRV1 has any significance *in vivo*.

A quenched fluorescent-tagged Dabcyl-QIDRIMEK-Glu(Edans)-NH_2_ peptide was used previously to measure the proteolytic activity of ASPRV1 *in vitro* (21). The QIDRIMEK sequence was identified as an effective target sequence of the PR using a library screening assay (21), but the cleavage position remains to be determined. Based on BLAST analysis, the synaptosomal-associated protein 25 (SNAP25) is the only human protein that contains a sequence showing 100% sequence identity to the QIDRIMEK peptide sequence. Different viral PRs were found to have the ability for cleaving such proteins in the infected cells which contain sequences being identical or highly similar to their autoproteolytic cleavage sites within the viral polyproteins (65, 66, 67, 68). A synthetic peptide representing the QIDRIMEK sequence was efficiently processed by ASPRV1, which implies that the SNAP25 protein is a candidate substrate of ASPRV1. In accordance with this, the target sequence is possibly accessible in SNAP25, based on the crystal structure of the complex of botulinum neurotoxin and SNAP25 (69). Nevertheless, it needs to be experimentally verified whether SNAP25 or similar proteins are substrates of ASPRV1 *in vivo*, although, SNAP25 and ASPRV1 are not coexpressed in the same tissue based on the Human Protein Atlas. Identification of additional substrates may reveal yet unknown biological functions of ASPRV1.

In regard to the patterns of cleavage site sequences, the specificity of ASPRV1 is similar to that of HIV-1 PR and other retroviral PRs which do not have a consensus cleavage site sequence, rather, the target sequences are diverse and the specificity is strongly sequence context-dependent (15, 63, 64, 70). In addition, the efficiency of the binding of the substrates to the retroviral HIV-1 PR is influenced not only by the sequence of the substrate but also by the volume (space) filled by the target site, as well. The mechanism that is based on the recognition of a substrate envelope has been described for HIV-1 PR (71, 72, 73) and for other viral PRs, as well, such as SARS-CoV-2 (74). Presumably, the mechanism of substrate recognition of ASPRV1 may be similar to that of the HIV-1 PR, and is potentially determined by a conserved substrate shape (75).

## Inhibition of ASPRV1

The eukaryotic retroviral-like PRs—that share high structural similarity with those of retroviruses (40)—are considered to be potentially inhibited by the PR inhibitors that are used in antiretroviral therapy of HIV-infected people. ASPRV1 was also thought to be susceptible to HIV-1 PR inhibitors, inhibition of which was supposed to potentially cause cutaneous side effects in the treated patients (1). Of the therapeutic antiretrovirals only indinavir was found to be capable of inhibiting ASPRV1 (1, 18), while other Food and Drug Administration-approved inhibitors (tipranavir, saquinavir, nelfinavir, darunavir, lopinavir, amprenavir (18), and ritonavir (1)) showed no inhibitory potential. It is important to note that indinavir has been discontinued and is not recommended for use by HIV/AIDS medical practice guidelines (https://hivinfo.nih.gov/). Pepstatin A and acetyl-pepstatin are potent inhibitors of numerous aspartic PRs but only moderate inhibitory potential was observed for ASPRV1, compared to indinavir (18). ASPRV1 may therefore potentially be inhibited by indinavir if administered as part of antiretroviral therapy, however, most Food and Drug Administration-approved HIV PR inhibitors have now been rendered ineffective due to the emergence of resistance-inducing mutations. Although, direct correlation was not found between cutaneous side effects and the inhibition of ASPRV1 PR by indinavir, its inhibition may contribute to the development of other side effects in other cell types where ASPRV1 has yet unidentified functions.

Natural resistance is not a unique feature of ASPRV1, other retroviral-like PRs were also found to be insensitive for most clinically used HIV-1 PR inhibitors, such as the yeast Ty1 retrotransposon PR (33), the human PEG10 PR (34), as well as the Ddi1PR (76) and Ddi2 PR (77, 78). A comparison of ASPRV1 and HIV-1 PR sequences revealed that multiple ASPRV1 residues correspond to some major or minor resistance mutations of HIV-1 PR in equivalent positions (18). However, it is important to note that natural resistance can hardly be interpreted solely at the level of primary protein structure, due to the overall differences between the tertiary and quaternary structures of retroviral and retroviral-like PRs. Detailed comparative analyses to identify the common characteristics of retroviral-like PRs that are responsible for this natural resistance are lacking.

To date, no specific inhibitors have not been designed specifically against retroviral-like proteins; despite being important targets. PEG10 is the first Gag-like protein for which inhibitors are being developed, and a patent for the use of these molecules is now pending (79). The target protein domain(s) and the rationale behind the inhibitor design are still unknown. The high structural similarity implies that ASPRV1, and other retroviral-like protease domain-containing proteins such as Ddi1 and Ddi2 may also be potentially inhibited by the protease inhibitors of PEG10. Therefore, if possible, it will be important to determine the potential interference of PEG10 inhibitors with ASPRV1.

## Protein variants

**Figure 5:**
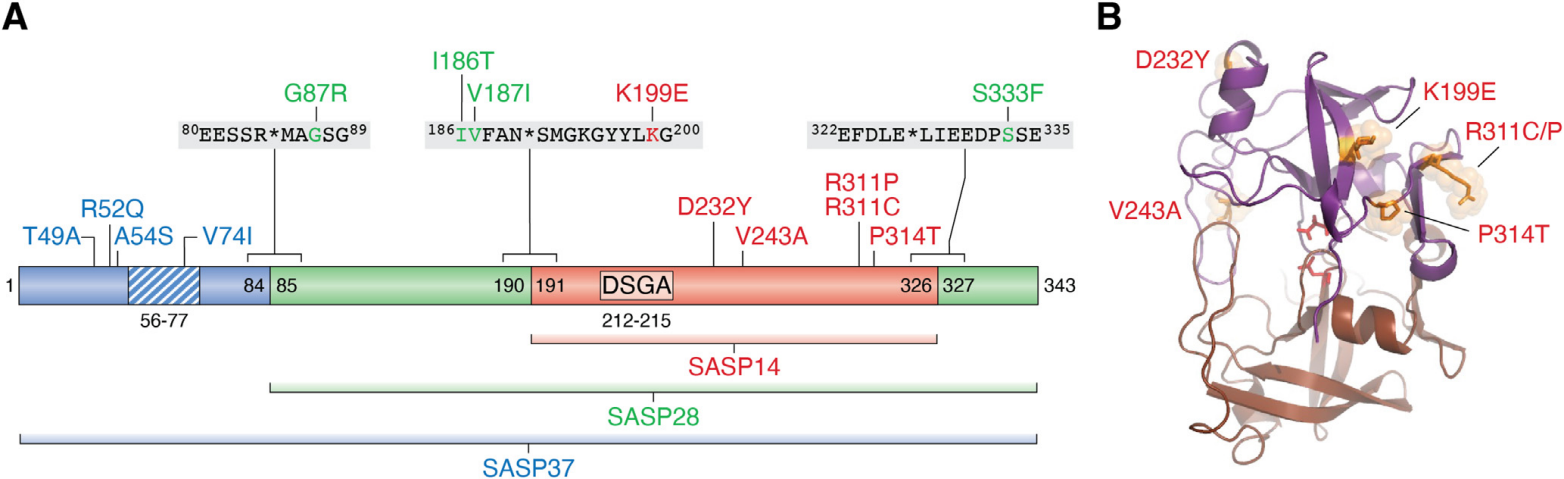
**Sequence variations of human ASPRV1.***A*, the schematic domain organization of ASPRV1 is represented. Some selected missense mutations and domain boundaries are labeled. The hydrophobic region is *striped*, and the D-S-G-A catalytic motif is also shown. The mutations which affect self-processing sites are shown together with the cleavage site sequences. The plotted mutations are listed in Table 2. *B*, mutations of the protease domain are shown in the homology model structure of ASPRV1-14, the catalytic aspartates are represented by *red sticks* (18). ASPRV1, retroviral-like aspartic protease 1.

Multiple ASPRV1 sequence variants have been identified to date (Fig. 5*A* and Table 2). Some of these variants were identified by high-throughput analysis of various cancers such as prostate cancer (80), colorectal carcinoma (81) or liver cancer (82). These alterations can be considered as passenger rather than causative mutations, the effects of the mutations were not further investigated at protein level and their correlations with the phenotypes were also not studied. Therefore, discussion of the sequence variants is limited in this article mainly to mutations that were also investigated for their association with phenotypic manifestations. Such mutations were identified by sequencing of *ASPRV1* gene from subjects with skin disorders such as *ichthyosis* (7), atopic dermatitis (47), eczema, or clinically dry skin (83).

**Table 2:** Known protein variants of human ASPRV1

Variant	Affected part of the protein	Effect of mutation on protease function	Reference
T49A	N-terminal domain	Not determined	(83)
R52Q	N-terminal domain	Not determined	(83)
A54S∗	N-terminal domain	Not determined	(47)
V74I	Hydrophobic region	Not determined	(83)
G87R	ASPRV1-28 N-terminal autoprocessing site P2′	Not determined	(83)
I186T	ASPRV1-28 N-terminal autoprocessing site P5	Elevated autoprocessing	(47)
V187I	ASPRV1-28 N-terminal autoprocessing site P4	Decreased autoprocessing	(47)
K199E	ASPRV1-14 N terminus (autoprocessing site P9′)	Impaired filaggrin processing	(7)
D232Y	ASPRV1-14 core	No remarkable effect on autoprocessing	(47)
V243A	ASPRV1-14 core	Loss of autoprocessing ability	(47)
R311P	ASPRV1-14 C terminus (autoprocessing site P16)	Impaired filaggrin processing	(7)
R311C∗	ASPRV1-14 C terminus (autoprocessing site P16)	Elevated autoprocessing	(47)
P314T	ASPRV1-14 C terminus (autoprocessing site P13)	Impaired filaggrin processing	(7)
S333F	ASPRV1-14 C-terminus (autoprocessing site P7′)	Not determined	(83)

The mutations are represented in Figure 5, as well. ∗ These mutations were identified in the same patient with atopic dermatitis and in the same allele.

Effects of the mutations were studied at the level of *cis*- and/or *trans*-activity of ASPRV1, *i.e.* in the case of its activation *via* self-proteolysis and/or filaggrin processing (Table 2). The amino acid substitutions within (V74I) or in the close proximity of the hydrophobic region (R52Q and A54S) was not investigated *in vitro*, due to the insufficient expression of ASPRV1-37 in bacterial expression system (1). Deleterious mutations of the CA-like domain were not reported so far, therefore, the effects of these mutations on the functions of this domain; such as intermolecular interactions, are currently unknown. Mutations were described for autoproteolytic cleavage site sequences, as well (G87R, I186T, and V187I) (Fig. 5*A*), these residues do not constitute a part of the CA-like domain, thus, their impact was investigated only at the level of the PR domain (47).

Mutations of the protease domain affect surface-exposed residues that are not buried in the hydrophobic core (Fig. 5*B*). The D232 residue is located near the additional helical insert, and the K199 is located in the proximity of the N terminus of the protease domain. The V243 residue is located within a flap that covers the active site, this residue contributes to ligand binding and it is a part of the S2 binding site (18). The P314 and R311 residues are close to the C terminus, the R311 is located within the last β-strand of the dimer interface. Due to the exposition of a cysteine side-chain to the enzyme surface, this residue might be susceptible to oxidation in the R311C mutant.

Mutation of the R311 residue (R311C) was found to increase the *cis*-activity, leading to elevated self-proteolysis. This mutation was identified in an atopic dermatitis patient along with A54S mutation, the effect of the latter mutation has not yet been determined (47). Interestingly, another mutation in the 311th position (R311P) decreased the *trans*-activity; *i.e.* resulting in impaired filaggrin processing. Besides R311P, the K199E and P314T mutations were also found to abolish filaggrin proteolysis, contributing to the development of *ichthyosis* (7). Self-processing site mutations were also identified in the case of each main autoproteolytic cleavage site and their close vicinity, such as G87R, I186T, V187I, K199E, and S333F (Fig. 5). Interestingly, the I186T mutation improved the autoprocessing ability while V187I mutation remarkably decreased the activity of the activity (47), and the K199E mutation caused impaired filaggrin processing (7).

A cleavage site mutation was also identified at the C terminus of ASPRV1 in a German Shepherd dog with *ichthyosis*, as well. The L351P mutant dog protein (L325P according to human ASPRV1 numbering) contained the mutation of the C-terminal self-processing site of ASPRV1-14. Replacement of the highly conserved leucine residue at the P2 position of the cleavage site was supposed to result in aberrant filaggrin processing and consequently contribute to the development of *ichthyosis* (84). Not only a point mutation but a frameshift mutation was also identified as a cause of ASPRV1 deficiency. Kiener *et al.* identified a heterozygous frameshift variant of *ASPRV1* gene in a Pembroke Welsh Corgi with *ichthyosis* (85). Due to a two base-pair deletion, the L199Rfs∗342 frameshift mutation was predicted to result in remarkable change of the protein sequence, such as considerable elongation of the protein as a result of the mutation (the WT and mutant proteins consist of 381 and 539 residues, respectively). Subsequently, 48% of the protein sequence was also predicted to be altered, most likely causing loss of ASPRV1’s function.

## Posttranslational modifications and regulation of ASPRV1

The full-length ASPRV1 undergoes posttranslational modifications (PTMs). The most important PTM is the autoproteolysis that releases the ASPRV1-28 and then the ASPRV1-14 forms. The self-processing of ASPRV1 causes autoactivation of the PR domain and is a well-established process, which has been described in this article above.

Other modifications, such as phosphorylation has not yet been studied in detail. Based on the PhosphoSitePlus database (86), ASPRV1 is phosphorylated at least at the 196th and 197th positions. These Tyr residues are located in the proximity of ASPRV1-14’s N terminus and correspond to the P6′ and P7′ residues of the N-terminal autoproteolytic cleavage site of the protease domain, respectively.

Although the effects of phosphorylation on the activity of ASPRV1 protease activity have not yet been explored, the regulation of ASPRV1-mediated filaggrin processing *via* phosphorylation is well-established (59). The pro-FLG is produced as an extensive phosphorylated precursor protein by granular keratinocytes. Upon epidermal differentiation, the pro-FLG undergoes dephosphorylation, followed by its cleavage into multiple filaggrin monomers that are abundant in the first layer of *stratum corneum*; the proteolytic processing is catalyzed by various proteases including ASPRV1 (59, 61, 62, 87). The pro-FLG is phosphorylated at multiple sites, both the FLG repeat units and the linkers may undergo this modification which is known to prevent the premature association of pro-FLG with keratin as well as the early maturation *via* proteolysis. Phosphorylation at the linker sequences—that connect the FLG units and comprise ASPRV1 cleavage sites—was found to prevent premature processing of the precursor protein (59). The P6-Ser (86), the P4-Ser (88), the P1-Tyr, the P3′-Ser, and the P4′-Thr residues (86) of the ASPRV1 cleavage sites in pro-FLG (SGSFLY↓QVSTH) can be phosphorylated, but the effects of the phosphorylation on the proteolysis was experimentally investigated only in the case of P4-Ser residue. Phosphorylation of Ser at P4 position was found to prevent the hydrolysis while the nonmodified peptide representing the same cleavage site was processed efficiently by ASPRV1-14 (18). As phosphorylation of the substrate (at least at P4 position of the filaggrin cleavage site) can prevent proteolytic processing, phosphorylation at P6′ or P7′ positions may also potentially contribute to the regulation of autoproteolysis. This is most likely if ASPRV1 PR contains a substrate-groove interaction surface, which would enable it to recognize not only P4-P4′ but also P12-P12′ substrate residues. Nevertheless, K199E mutation—at P9′ site of the N-terminal autoproteolytic site of ASPRV1-14—was reported to cause loss of enzyme activity (7). The effects of the cleavage site-phosphorylation (*e.g.* at P6′ or P7′ positions) and -mutation (*e.g.* K199E mutation at P9′ position) can be investigated in the future in order to determine whether they induce conformational changes of the enzyme or affect the interactions with the sequence to be cleaved during self-activation.

Based on the most recent findings, activity of ASPRV1 can be regulated not only by PTMs but also by protein-protein interactions. FLG2 was identified as an interaction partner of ASPRV1 (especially that of the 28 kDa protein form). Both proteins are partially colocalized at the *stratum granulosum*, and the interaction between them is mediated by their so-called SID domains (2–95 and 12–84 regions in FLG2 and ASPRV1, respectively) (21). The SID domain of ASPRV1 corresponds to its CA-like domain (97–169 region), its interaction with the N-terminal domains of FLG2 improves the protease activity of ASPRV1, enhancing autoactivation *in vitro.* The molecular mechanism behind the activation remains to be determined (21), future studies are expected to reveal whether interaction-induced conformational changes or the release of the N-terminal domain triggers the activation. Nevertheless, interaction with FLG enhances the self-activation of ASPRV1, improving its *trans*-activity, thus, resulting in elevated processing of FLG to natural moisturizing factors. Consequently, the functional importance of the interaction between FLG2 and ASPRV1 is to fine-tune the epidermal terminal differentiation and the moisturization of the epidermis *via* regulation of FLG maturation (21).

## ASPRV1 expression and clinical correlations

ASPRV1 has been identified as a mammalian-specific gene originating from the insertion of a retroviral gene or the transposition of a retroviral element into an ancestral mammalian genome (89, 90). The expression of ASPRV1 in the stratified epidermis was found to be unique to mammals (eutherians, marsupials, and monotremes) (12, 91). In accordance with this, the expression of pro-FLG—which is the physiological substrate of ASPRV1—in the skin is also specific for mammals, consequently, it is likely that pro-FLG and ASPRV1 contribute to mammalian-specific features of the barrier system of the skin (12). Besides ASPRV1, caspase 14 also contributes to FLG processing by breaking down the monomeric FLG units, and this enzyme is expressed in many species together with ASPRV1 and pro-FLG (including as in humans).

Interestingly, terrestrial and fully aquatic mammals exhibit differences in the conservation of the *ASPRV1*, *caspase 14*, and *pro-FLG* genes. Some mammals lost *caspase 14* during the evolution (such as dolphins) while some others (cetaceans such as minke and sperm whales) lost both *pro-FLG* and *caspase 14*, but *ASPRV1* is conserved in all of these species (89). Neutrophils also express ASPRV1 but not pro-FLG (8). The conserved expression of *ASPRV1* without that of *caspase 14* and *pro-FLG* implies functions of ASPRV1 that are independent from the proteolytic processing of pro-FLG, such as proteolysis of yet unidentified substrate or mediation of protein-protein interactions.

The role of ASPRV1 in the maintenance of epidermis is well-established, based on the correlation of ASPRV1 expression with some skin-related diseases, and by studies on KO mice. Transiently elevated ASPRV1 expression was observed during skin carcinogenesis of mice (39). Transgenic KO mice showed fine skin wrinkles, although no defects of epidermal differentiation were observed that implied that the protease activity of ASPRV1 contributes to the organization of skin tissue (2). Some stress conditions such as phorbol ester treatment were found to induce the expression of ASPRV1 (39), while overexpression of ASPRV1 resulted in delayed wound healing but not altered keratinocyte proliferation or aberrant differentiation, indicating a possible effect of ASPRV1 on keratinocyte migration and a functional importance in skin regeneration (11). ASPRV1-deficient hairless mice showed more fine wrinkles and more dry and rough skin, and the *stratum corneum* was thicker and showed decreased hydration. This phenotype was associated with impaired pro-FLG processing, causing its accumulation, but no changes of the free amino acid composition was noticed, hence proving the role of ASPRV1 in pro-FLG processing and in the hydration of *stratum corneum* (47). Although, multiple missense mutations were identified in the *ASPRV1* gene of patients with atopic dermatitis, and some of them were found to abolish the protease activity (47), no evidence was found for the direct association between the ASPRV1 mutations and dry skin, at least in the context of European population (83). Interestingly, some of other mutations that were identified in dogs (84, 85) and humans (7) showed correlation with *ichthyosis vulgaris* (Table 2).

Several studies confirmed that ASPRV1 is expressed in various cell types, and proteomic analyses as well as gene expression profiling studies revealed changes of ASPRV1 in different conditions and diseases (Table 3). These data support the hypothesis that ASPRV1 may have a function in other tissues apart from the skin, but neither physiological nor pathophysiological importance of ASPRV1 has been yet revealed. For example, substrates of ASPRV1 PR in other cell types are still unknown. The changes of ASPRV1 expression in various cancer types along with the identified non-synonymous variants causing mutation of the protein are available in the TissGDB (Tissue specific Gene DataBase in cancer) database (92). Based on PhosphoSitePlus database (86) ASPRV1 mutations can be detected in tumor samples, but only with very low frequency, the highest frequency was found in colorectal, endometrial, and squamous lung is <1.5%, indicating no oncogenic potential for ASPRV1 mutations.

**Table 3:** Studies that revealed changes of ASPRV1 expression

Disease/Physiological process	Change of ASPRV1 expression	Species	Comment	Reference
Triple-negative breast cancer	Upregulated	Human	ASPRV1 was one of the top50 upregulated genes of in the Kenyan triple-negative breast cancer (TNBC) samples as compared to those of African-Americans and Caucasians	(103)
Atopic dermatitis (AD)	Upregulated	Human	Meta-analysis of AD datasets, ASPRV1 is one of the most upregulated epidermal proteases	(104)
Human papillomavirus (HPV)-negative head and neck squamous cell carcinoma (HNSCC)	Upregulated	Human	Differential expression as compared to HPV-positive HNSCC	(105)
Uninflamed acute respiratory distress syndrome (ARDS)	Upregulated	Human	Upregulated in blood leukocytes as compared to “reactive” phenotype	(106)
Colorectal cancer (CRC)	Upregulated	Human	Investigation of MACC1 (metastasis associated in colon cancer 1)-induced changes in SW480 human colon adenocarcinoma cell line (top 50)	(107)
*Stratum corneum* photodamage	Overexpressed (protein)	Human	Based on the comparison of corneomes *stratum corneum* samples of photodamaged cheek and photoprotected postauricular sites in Caucasian women	(108)
Lichen planus (chronic mucocutaneous disease)	Upregulated	Human	Comparison of oral lichen planus (OLP) and genital lichen planus (GLP) epithelium to normal oral and genital epithelium, respectively (top 20)	(109)
SARS-CoV-2 infection	Downregulated	Human	ASPRV1 is downregulated in SARS-CoV-2 infected human normal bronchial epithelial cell line as compared to mock-treated cells	(110)
Kawasaki disease (KD)	Downregulated	Human	Downregulated in KD subjects with coronary artery aneurysms as compared to normal coronary arteries	(111)
Serum protein	-	Human	ASPRV1 was identified as a part of serum proteome	(97)
Primary immune thrombocytopenia	-	Human	Coexpressed with genes having immune function-related genes	(112)
Rheumatoid arthritis (RA)	-	Human	ASPRV1 was one of the genes that was found to be related to the efficacy of methotrexate in RA therapy (based on text-mining) (top 24)	(113)
lung adenocarcinoma	Upregulated	Human	Coexpression with CYP3A43	(114)
nasal epithelial lining fluid	-	Human	ASPRV1 is one of the extracellular proteases identified in nasal epithelial lining fluid	(24)
Haematopoiesis	-	Mouse	ASPRV1 is highly expressed by neutrophils based on mouse RNA-seq data obtained from Haemopedia	(115)
Medullary thymic epithelial cells	-	Mouse	Reproductive tissue-restricted antigens highly expressed in mature medullary Thymic epithelial cells in the mouse (umbilical cord)	(116)
Trophoblast stem cell differentiation	-	Mouse	ASPRV1 exon was identified as a novel exon in trophoblast cells	(117)
Fibrosis	-	Mouse	ASPRV1 expression in fibrosis-associated macrophages	(118)
Cell differentiation	Upregulated	Mouse	Ca^2+^-induced protein expression in keratinocytes (top 5)	(119)
Lung development	Upregulated	Mouse	High ASPRV1 expression in neutrophils (live CD45+ lung cells) (top25 DEG)	(120)
JHM strain of mouse hepatitis virus (JHMV)-infection (demyelination)	Upregulated	Mouse	CNS-infiltrating neutrophils (isolated from spinal cord)	(121)
Type 1 diabetes	Upregulated	Mouse	Bone marrow neutrophils from streptozotocin-treated mice (top6 DEG)	(122)
BCG (Bacille Calmette Guérin) challenging	Upregulated	Mouse	ASPRV1 is differently expressed in macrophages of BCG-challenged and control mice (top 5 DEG)	(123)
Oxidative stress (peroxide stimulation)	Upregulated	Mouse	Studies on murine embryonic fibroblast cells that express WT or redox-insensitive mutant Stat3	(124)
Diabetes mellitus type 2	Upregulated	Mouse	Bone marrow cells	(125)
^131^I irradiation	Upregulated	Mouse	Upregulated in lung, and spleen as compared to nonirradiated controls	(126)
Inflammatory skin disease	upregulated	Mouse	Upregulated in RAC1-null epidermis (RAC1-null keratinocytes) as compared to control (noninflammatory)	(127)
Chemically induced colitis-associated cancer (CAC)	Upregulated	Mouse	Colon tissues from control, azoxymethane (AOM) and AOM/dextran sulfate sodium salt (DSS)-treated mice	(128)
Prostate cancer	Upregulated	Mouse	*ASPRV1* gene is responsive NF-κB	(129)
Cellular stress	Downregulated	Dairy cow	Differential hepatic expression in cows with high and low fibroblast growth factor 21 (FGF21) expression	(130)
Equine squamous gastric disease (ESGD)	Upregulated	Horse	The ASPRV1 is a serum protein markers for ESGD	(95)

For comparison, data from species other than human are also shown. Data are shown based on literature data. DEG, differentially expressed gene; PI, protease inhibitor. If ASPRV1 was in the top DEGs, it is indicated in parentheses.

The findings of multiple studies support the putative importance of ASPRV1 in cell types having immune function such as neutrophils and macrophages (8, 93, 94). In horses, the ASPRV1 was found to be a serum protein marker of both mild and moderate equine squamous gastric disease, as compared to the serum samples of the horses that were nondiseased (95). Based on the data available in Plasma Proteome Database (96) as well as in EVpedia, ExoCarta, and Vesiclepedia databases (97), ASPRV1 is part of the human serum proteome, but its pathological and physiological importance in the human serum remain to be elucidated. In addition, ASPRV1 was found to be part of the sweat proteome (98) and is present in the nasal epithelial lining fluid (24), indicating extracellular functions.

## Summary

ASPRV1 belongs to the family of the Gag-like proteins which originated from retroelements and have been domesticated during the mammalian evolution. ASPRV1 was identified first in human skin where it was found to contribute to moisturization *via* processing of pro-FLG, which appears to be its only physiological substrate. Some mutations of ASPRV1 were found to impair its proteolytic activity and result in the accumulation of unprocessed pro-FLG, even causing a deficiency of skin hydration (ichthyosis) (7). These mutations are classified into the group of the ASPRV1-associated causative factors of the autosomal dominant lamellar ichthyosis (ADLI) (99), and belong to the group of epidermis disorders being associated with abnormal proteolytic activity (100). Several biochemical characteristics of ASPRV1 have already been determined, but the currently available data imply that it might potentially have yet unknown physiological functions. The mechanisms used for the activation from the precursor protein and for substrate recognition (*i.e.* substrate envelope) also resemble those of viral PRs, such as HIV-1 and SARS-CoV-2. ASPRV1 was found to be able to process some protein substrates, such as its natural substrate pro-FLG as well as the general protease substrates insulin and casein. Its ability to cleave peptides representing sequences of HIV-1 polyprotein and SNAP25 human protein implies that ASPRV1 is potentially capable of processing yet unidentified targets, even in the extracellular space such as in sweat (98), serum (95), and airway fluid (24). Identification of additional extra- and/or intracellular substrates may significantly aid in better understanding of the specificity of the protease. In addition, identification of new substrates is expected to reveal yet unknown physiological functions that are not limited to the skin (91), and may uncover its role at least in immune cells (8, 93, 94). Molecules for specific inhibition of retroviral-like PRs are not currently available, therefore, the determination of the structural requirements of substrate recognition (*e.g. via* substrate envelope and substrate-groove) can provide valuable information for *in silico* drug design. The candidate protease inhibitors to be developed *e.g.* against PEG10 (79) may potentially inhibit enzymes that share high structural similarity with it, such as ASPRV1. The identification of the features of other retroviral-like proteins, such as PEG10 (4, 46, 101) as well as the large-scale comparison of the domesticated retroelement-derived genes (3, 4, 5, 13, 25, 102) is also expected to help in exploring the characteristics of ASPRV1. Transcriptomic and proteomic analyses have already revealed expression of ASPRV1 in various cell types other than the skin. Therefore, we recommend the use of ASPRV1 names rather than SASPase, and accordingly we propose using the name ASPRV1-37, −28, and −14 to distinguish the precursor and mature protein forms. Altered ASPRV1 expression was also observed in multiple diseases, mainly transcriptomic analyses revealed these changes (upregulation in most cases); thus, determination of the changes in the level of ASPRV1 may provide more relevant information on its contribution to pathological phenotypes. Comparative analysis of retroviral-like proteins is also expected to reveal common and unique biochemical features.

## Conflicts of interest

The authors declare that they have no conflicts of interest with the contents of this article.
